# Lung ultrasound-guided protocolized fluid management versus conventional central venous pressure-based management in monitoring extravascular lung water during the perioperative period of percutaneous nephrolithotomy: a randomized controlled trial

**DOI:** 10.1080/07853890.2026.2741437

**Published:** 2026-10-09

**Authors:** Xuan Yu, Zhiwei Wang, Xinpeng Xu, Yu Shuai, Wen Cheng, Zhao Wang, Yi Zhang

**Affiliations:** Department of Anesthesiology, The Second Affiliated Hospital of Zunyi Medical University, Zunyi, China

**Keywords:** Lung ultrasound, central venous pressure, extravascular lung water, percutaneous nephrolithotomy, fluid management

## Abstract

**Background:**

Percutaneous nephrolithotomy (PCNL) is a common surgical intervention to treat renal calculi. Excessive irrigation fluid absorption during surgery may enhance extravascular lung water (EVLW) level. Hence, accurate monitoring and protocolized management of EVLW are crucial for preventing postoperative complications.

**Methods:**

Our randomized controlled study enrolled 181 cases undergoing elective PCNL and randomly divided them to conventional CVP-based fluid management (Group C) or lung ultrasound-guided protocolized fluid management (Group LUS). Primary outcomes included increased incidence of EVLW and severity within 30 min postoperatively and postoperative hypoxemia in the two groups. Secondary outcomes were the CVP values and LUS scores at the following time points: before irrigation (T0), at the first administration of furosemide (T1), after irrigation (T2), and at the time of leaving the post-anesthesia care unit (PACU) (T3). Moreover, included fluid volumes (crystalloid, colloid, total infusion, irrigation, and supplemental fluid at first furosemide administration), total furosemide dosage. The anesthesia time, surgical duration, PACU stay time, extubation time, and total hospital stay were recorded. Safety outcomes included perioperative complication rates.

**Results:**

Compared with Group C, Group LUS demonstrated significantly lower incidence and severity of EVLW increase within 30 min postoperatively, as well as reduced postoperative hypoxemia rate (all *p* < 0.001). Group LUS displayed evidently lower LUS scores and CVP values at T1、T2 and T3 than Group C (*p* < 0.001). Moreover, extubation time and PACU stay in Group LUS were significantly reduced, compared to Group C (*p* < 0.05).

**Conclusions:**

An LUS-guided fluid management protocol integrating real-time EVLW assessment with predefined intervention thresholds outperformed conventional CVP-based management during the perioperative period of PCNL. The superior sensitivity of LUS for detecting early EVLW elevation, combined with protocolized early intervention, reduced postoperative hypoxemia incidence, shortened extubation time and PACU stay duration, and improved short-term perioperative recovery.

## KEY MESSAGES

LUS-guided protocolized fluid management enables earlier detection of EVLW elevation than conventional CVP-based monitoring, allowing timely intervention during PCNL.

Protocolized LUS-guided management significantly reduces postoperative EVLW severity and hypoxemia risk, while shortening extubation time and PACU stay duration.

Integrating LUS as a non-invasive real-time monitoring tool within a structured management protocol optimizes perioperative fluid management and improves short-term recovery after PCNL.

## 1. Introduction

As a minimally invasive surgery, percutaneous nephrolithotomy (PCNL) has been frequently used for managing renal calculi [1]. To ensure a clear surgical field, irrigation fluid is required to irrigate the stone sites, such as the renal pelvis and calyces [2]. However, excessive absorption of irrigation fluid enhances the body’s fluid load, thereby raising the hydrostatic pressure in the alveolar capillaries and promoting the production of extravascular lung water (EVLW) [3]. This might induce pulmonary edema and even lead to heart failure, worsening the patient’s condition. Therefore, anesthesiologists should promptly identify and address the potential adverse effects of increased perioperative EVLW on various body systems to ensure the patient’s intraoperative anesthesia safety. Hence, the objective monitoring, accurate prediction, and assessment of the body’s volume status are critical in clinical anesthesia practice.

Traditionally, the anesthetic management of PCNL has been based on monitoring of Central Venous Pressure (CVP) and estimation of irrigation fluid volume to indirectly assess the volume status [4]. However, CVP can only indirectly reflect the right ventricular preload. Being susceptible to the influence of body position, cardiac function, and intrathoracic pressure, it requires invasive catheterization and cannot quantify real-time changes in lung water [5]. Moreover, a large error in estimating irrigation fluid absorption volume can easily cause volume overload and progression to pulmonary edema [6]. Additionally, all current clinical methods for assessing EVLW have their limitations. Physical examination and auscultation have poor accuracy, and chest X-ray exhibits low sensitivity. Although accurate, a CT scan cannot be performed at the bedside in real time, while the transpulmonary thermodilution technique, despite its accuracy, is invasive and not common [7–9].

In recent years, lung ultrasound (LUS) has gradually attracted attention due to its advantages, such as non-invasiveness, convenience, and strong repeatability [10,11]. LUS can semi-quantitatively assess EVLW alterations through evaluating B-line number and distribution [12], conducive to an early diagnosis and treatment of intraoperative pulmonary edema. However, very few studies have been conducted on LUS during PCNL.

Consequently, this study aimed to evaluate a protocolized fluid management strategy guided by LUS, in which predefined ECS thresholds trigger early intervention, and compare it with conventional CVP-based management during PCNL. We hypothesized that integrating LUS’s superior sensitivity for EVLW detection with a structured intervention protocol would improve perioperative outcomes compared with reactive management based on CVP thresholds and clinical judgment.

## 2. Materials and methods

### 2.1. Patient enrolment

The Ethics Committee of Second Affiliated Hospital of Zunyi Medical University approved our study protocol (KYLL-2022-057). This study was registered at the Chinese Clinical Trial Registry on September 5, 2023 (Registration Number: ChiCTR2300075416). URL: https: www.chictr.org.cn/showproj.html?proj=201155. Patient enrollment commenced on October 10, 2023. Written informed consent was obtained from all participating subjects; for subjects unable to provide consent independently, informed consent was signed by their corresponding family members or legal representatives.

After pre-trial preparations from September to October 2023, we enrolled 181 patients undergoing elective PCNL at the Second Affiliated Hospital of Zunyi Medical University between October 2023 and December 2024. Our inclusion criteria included: patients aged >18 years; those with Body Mass Index (BMI) of 18.5–28 kg/m^2^; participants with ASA Physical Status Classification I-II; those receiving elective PCNL, and participants whose preoperative chest X-ray or chest CT showed no abnormalities. The exclusion criteria were: presence of severe cardiac, hepatic, or pulmonary insufficiencies; recent history of upper respiratory tract infection; those with an abnormal coagulation function; participants whose surgery delayed for more than 24 h; those who were postoperatively transferred to ICU for treatment; subjects unable to cooperate with follow-up or poor compliance; those with loss or unavailability of crucial data, and participants who withdrew for any reason.

### 2.2. Anesthetic procedure

Preoperatively, patients fasted for eight and two hours from food and water, respectively. After entering the operating room, their upper limb veins were cannulated, accompanied by monitoring of their non-invasive blood pressure, electrocardiogram (ECG), as well as pulse oxygen saturation (SpO_2_). Additionally, LUS assessment and scoring were performed before anesthesia. The anesthesia induction protocol was uniformly standardized. To induce general anesthesia, midazolam (0.05 mg/kg), etomidate (0.2–0.3 mg/kg), sufentanil (0.2–0.3 μg/kg), alongside rocuronium (0.8 mg/kg) was used. Post-anesthesia induction, both groups underwent endotracheal intubation, with ventilation in volume-controlled mode. The ventilator parameters were controlled for maintaining PETCO_2_ at 35–45 mmHg. CVP monitoring was also completed through right internal jugular vein puncture and catheterization. Anesthesia maintenance was achieved by continuous intravenous infusions of propofol (50–100 μg/kg/min), remifentanil (0.05–0.15 μg/kg/min), and 2–4% sevoflurane (50% O_2_/air mixture). Postoperatively, each case with an endotracheal tube was transferred into Post-Anesthesia Care Unit (PACU). After extubation, patients underwent oxygen inhalation at 3 L/min *via* a face mask. After their Steward scores were ≥4, they were returned back to the ward.

### 2.3. Surgical procedure

The PCNL was performed by the same team from our hospital’s Department of Urology. Following an endotracheal intubation anesthesia, patients lay in the modified lithotomy position, and their waists as well as buttocks were lifted. After skin preparation, the ureteral orifice was examined by inserting a ureteroscope transurethrally. A 5 F ureteral catheter was placed on the affected side after retaining an indwelling urinary catheter. Post-injection of sterile normal saline to establish artificial hydronephrosis, the surgeon punctured the target calyx under ultrasound guidance, inserted a safety guidewire, and dilated the tract to 16 F/22F. Subsequently, a ureteroscope or nephroscope was inserted through the puncture sheath. Holmium laser or ultrasound with pneumatic lithotripsy was used for managing lithotripsy and stone extraction. Dual-tract lithotomy was performed if required. Post-lithotripsy, the ureteral catheter was removed, and a ureteral stent (5 F/6F) as well as a drainage tube were inserted, secured, and retained.

### 2.4. Lung ultrasound examination

We used the Mindray portable ultrasound device (Model M9 + dedicated convex array transducer). The EVLW assessment was based on Echo Comet Score (ECS) measured through the 28-intercostal space approach [13]. This approach involves scanning the 2^nd^-4^th^ and 2^nd^-5^th^ intercostal spaces on bilateral sides individually, along the midaxillary, anterior axillary, midclavicular, and parasternal lines, respectively. ECS comprises the overall B-line numbers detected within these 28 intercostal spaces. A B-line refers to the hyperechoic line originating from pleural line extending to the screen edge like a laser beam without attenuation, and moves with respiration [14]. The scoring criteria were based on the semi-quantitative B-line score [15]. Moreover, EVLW was classified according to the ECS scores: When the B-line number was ≤ 5, the score was 0, thereby indicating no EVLW; when the number was 6–15, the score was 1, suggesting mild EVLW; and when the number was 16–30, the score was 2, indicating moderate EVLW. However, when the B-line number was ≥31 or diffusely distributed throughout the lungs, it was classified as severe EVLW (Figure 1). All operators underwent ultrasound qualification training at our hospital and obtained formal certification.

**Figure 1.**
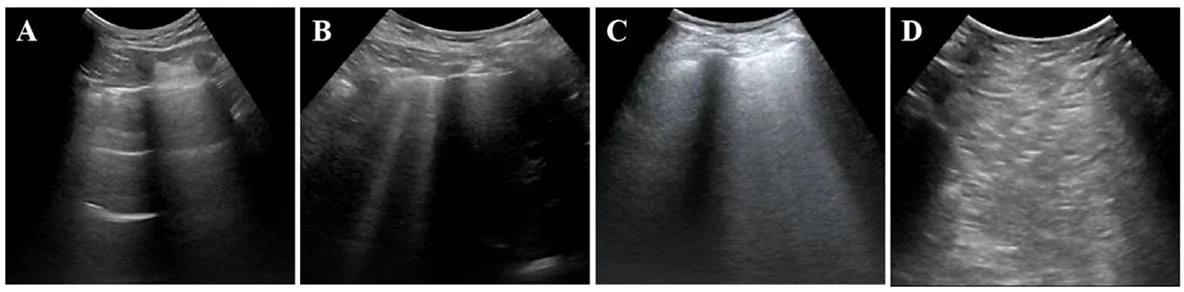
Lung ultrasound scoring criteria. (A) When B-lines ≤ 5, the score was 0, no EVLW was observed; (B) When B-lines were 6–15, the score was 1 and mild EVLW was suggested; (C) When B-lines were 16–30, the score was 2, indicating moderate EVLW; (D) B-lines ≥31 or diffusely distributed throughout the lungs suggested severe EVLW.

### 2.5. Grouping and intervention

We randomized patients into a conventional CVP-based fluid management or an LUS-guided protocolized fluid management group (Groups C and LUS), respectively, using a random number table method.

Group C (Conventional CVP-based management): The CVP was maintained between 5–12 cmH_2_O. This range served as the primary hemodynamic target, with intervention being reactive in nature. If the CVP exceeded the upper normal range limit, the anesthesiologist and surgeon jointly decided whether to administer a diuretic such as furosemide (0.3 mg/kg) or restrict infusions and irrigation fluids, based on the patient’s specific conditions, including vital signs, perfusion volume, and perfusion duration. Notably, this decision incorporated multifactorial clinical judgment without predefined EVLW-specific thresholds; CVP elevation represented a relatively late indicator of volume overload, often occurring after significant irrigating fluid absorption. In conditions of decreased oxygen partial pressure, hemodynamic instability, or life-threatening conditions, the surgeon suspended the surgery.

Group LUS (Protocolized LUS-guided management): Ultrasound examinations and scoring were performed at the incremental volume nodes of irrigating fluid (e.g. 100, 200, 300, 400, 500 ml/kg). Intervention was protocolized and triggered by predefined ECS thresholds: upon detection of mild EVLW (ECS score = 1, corresponding to 6–15 B-lines), clinical intervention was promptly initiated without awaiting hemodynamic compromise. This included administration of furosemide (0.3 mg/kg) and restriction of infusions, along with restriction of irrigating fluids. If subsequent ultrasounds showed a persistent increase in B-lines, additional doses of furosemide were administered (0.3 mg/kg). This approach thus integrated enhanced monitoring sensitivity with an early, structured intervention algorithm.

These two protocols differ not only in the monitoring modality employed (LUS vs. CVP) but fundamentally in management philosophy: proactive, threshold-driven intervention based on direct EVLW assessment versus reactive intervention based on indirect hemodynamic parameters and clinical judgment.

### 2.6. Observational indicators

Our primary outcomes included the increased incidence of EVLW and severity within 30 min postoperatively and postoperative hypoxemia (oxygen saturation <90%) in the two groups. Hypoxemia was defined as SpO_2_ < 90% lasting more than 10 s within 30 min postoperatively, or a PaO_2_/FiO_2_ ratio below 300 mmHg. The secondary outcomes were the CVP values and LUS scores at the following time points: before irrigation (T0), at the first administration of furosemide (T1), after irrigation (T2), and at the time of leaving the PACU (T3). Intraoperative fluid-related indices: crystalloid and colloid infusion volumes, total fluid infusion volume, total irrigation fluid volume, irrigation fluid volume and fluid replacement volume at the initial administration of furosemide, and total furosemide doses; time-related indices: anesthesia time (from its initiation to endotracheal extubation), operation duration, extubation time (between surgery completion and endotracheal extubation), PACU stay time (between PACU admission and safe PACU discharge), and total hospital stay. Incidence of perioperative complications included the following parameters: hypertension (an increase by 20% relative to baseline); hypotension (a reduction by 20% relative to baseline); tachycardia (heart rate >120 bpm); bradycardia (heart rate <50 bpm); hypothermia (core body temperature <36 °C); postoperative shivering observed as skeletal muscles’ involuntary rhythmic contractions; and pulmonary edema [10] (characterized by B-lines on LUS, i.e. ≥ 3 B-lines inside a single intercostal space or confluent B-lines occupying the majority of the intercostal spaces; combined with clinical manifestations like dyspnea, cough, expectoration (possibly with pink frothy sputum), chest tightness, etc. In severe cases, orthopnea and cyanosis might be observed, and moist rales as well as wheezes can be heard in both lungs on auscultation.

### 2.7. Randomization and blinding

Prior to anesthesia, patients were randomized 1:1 into Group C or Group LUS using a random number table generated by SPSS 26.0. Group allocation sequences were sealed in opaque envelopes and opened by an anesthesiologist who was blinded to group assignments. For intraoperative management, LUS examinations in the LUS group were performed by two anesthesiologists specialized in ultrasound who had obtained ultrasound qualifications; after reaching consensus on ECS scores, these two physicians directly informed the attending anesthesiologist of the results to guide clinical decision-making, and were thus necessarily unblinded to group allocation during the intervention phase. To minimize outcome assessment bias, a separate blinding procedure was established for postoperative evaluation: at 30 min postoperatively, two different anesthesiologists, distinct from the intraoperative ultrasound team and with equivalent ultrasound expertise and qualifications, performed LUS examinations on all patients from both groups. These outcome assessors were strictly blinded to group allocation and intraoperative management details, had not participated in any clinical decision-making during surgery, and independently scanned and scored all patients using the standardized 28-intercostal space protocol, with consensus reached for final ECS scores. This two-tier personnel structure ensures that outcome assessors were unbiased by knowledge of which management protocol each patient received, while preserving the real-time clinical utility of intraoperative LUS guidance.

### 2.8. Sample size estimation

According to findings from a preliminary pilot study, the incidences of increased postoperative EVLW in Groups C and LUS were 22% and 5%, respectively. Setting α = 0.05 and 1-β = 90% as the significance level and power, our sample size was calculated as 80 patients for each group using PASS 15 software. Considering a 20% dropout rate, 200 cases were included into this work, with 100 in each group.

### 2.9. Statistical analysis

SPSS 25.0 and R 4.5.1 software helped in statistical analyses. Normality and homogeneity of variance of continuous data were analyzed by the Shapiro-Wilk test and Levene’s test, respectively. Thereafter, normally-distributed data were represented by mean ± standard deviation (x̅±s) and compared by an independent samples t-test. Moreover, non-normal variables were represented by median (interquartile range) [M (IQR)] and compared by Mann-Whitney U test between groups. Multiple comparisons of all p-values were completed with the Bonferroni method. Categorical data were represented by a number (percentage) [n (%)] and compared by chi-square test (χ^2^ test) or Fisher’s exact test. For comparisons among ordinal variables, χ^2^ test, ordinal logistic regression, and Mann-Whitney U test were adopted.

## 3. Results

### 3.1. Baseline characteristics

Although we recruited 319 subjects, 200 subjects satisfied our inclusion criteria initially post-screening against the eligibility criteria. In Group C, two patients’ preoperative chest CT scans suggested suspected bronchitis or other abnormal findings, two patients exhibited B-lines during preoperative LUS scanning, the intraoperative surgical approach was changed for two patients, and the other two did not have postoperative follow-up data. Finally, 92 patients were included for analysis. In Group LUS, two patients had preoperative chest CT scans indicating suspected bronchitis or other abnormal results, two exhibited B-lines on preoperative LUS, six patients had an intraoperative change in surgical intervention, and one patient had lost postoperative follow-up data. Eventually, 89 subjects were enrolled for analysis. As the number of post-randomization exclusions was small and exclusion reasons were unrelated to treatment outcome, the per-protocol analysis was considered the most appropriate primary analysis (Figure 2).

**Figure 2.**
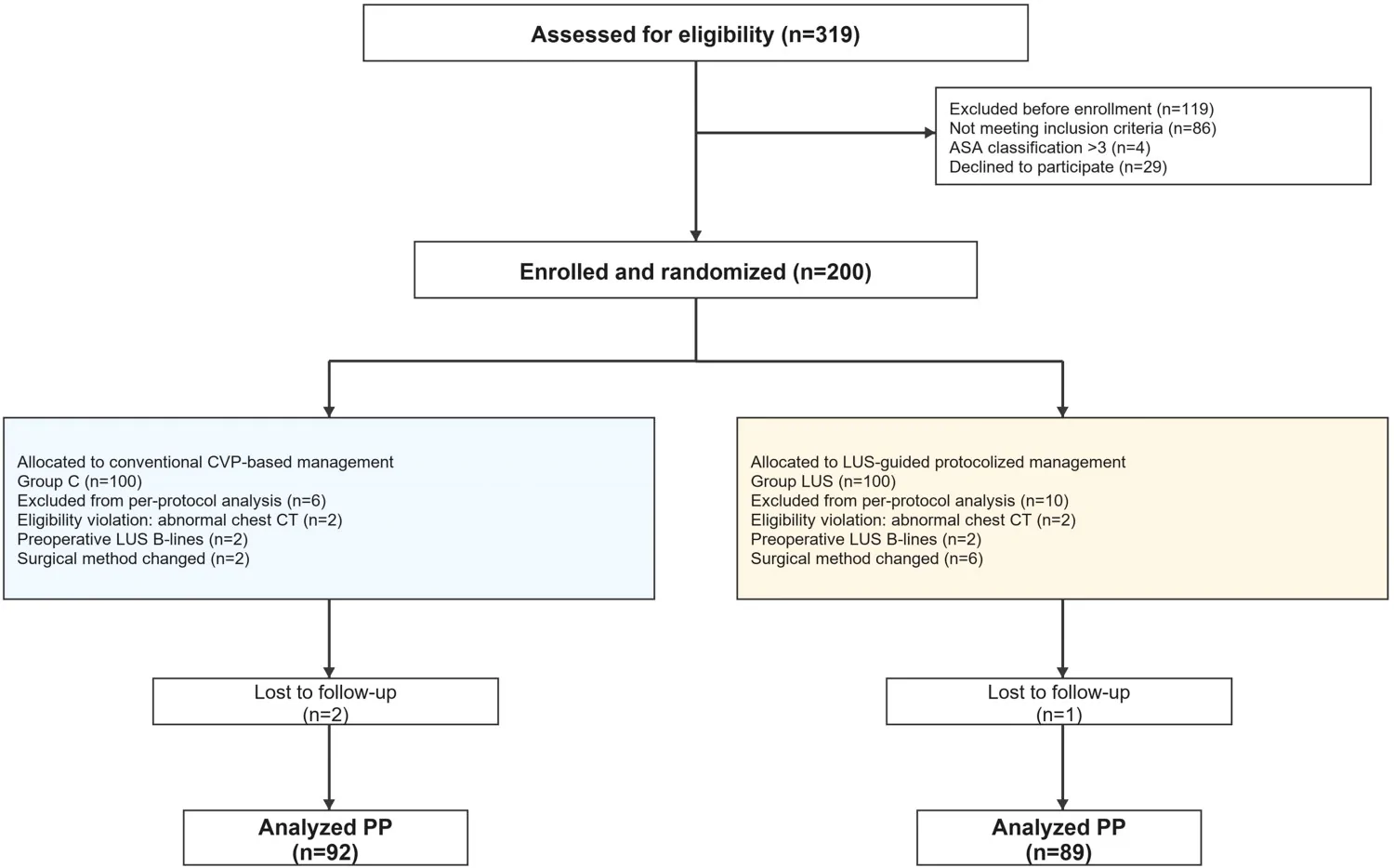
Flowchart depicting the study’s procedure baseline characteristics.

Group LUS had markedly reduced extubation (27.75 ± 9.28 min vs. 30.85 ± 7.86 min; *p* = 0.015) and PACU stay time (55 [IQR 11.50] min vs. 60 [IQR 11.75] min; *p* < 0.001) relative to Group C (*p* < 0.05). Various parameters like sex, age, height, weight, BMI, ASA classification, stone diameter and location, drug dosages during anesthesia induction and maintenance, preoperative hemoglobin (Hb), hematocrit (Hct), albumin, preoperative comorbidities, perioperative usage of vasoactive drugs, operation duration, anesthesia time, and total hospital stay did not show any significant difference between groups (*p* > 0.05) (Table 1).

**Table 1.** Baseline characteristics in two groups.

	Group C (*n* = 92)	Group LUS (*n* = 89)	*P*-value
Sex [n(%)]			0.12
Male	63(68.48)	51(57.30)	
Female	29(31.52)	38(42.70)	
Age (years)	56.54 ± 11.27	54.96 ± 11.52	0.35
Height (cm)	159.83 ± 4.41	160.12 ± 6.47	0.717
Weight (kg)	59.26 ± 5.29	61.02 ± 9.47	0.124
BMI (kg/m^2^)	23.16 ± 1.74	23.86 ± 3.12	0.061
ASA [n(%)]			0.346
I	35(38.04)	40(44.94)	
II	57(61.96)	49(55.06)	
Stone diameter (mm)	15(8.75)	17(8)	0.297
Stone location[n(%)]			0.714
Renal pelvis	29(31.52)	26(29.21)	
Renal calyx	43(46.74)	39(43.82)	
Upper ureter	20(21.74)	24(26.97)	
Anesthesia induction drugs			
Midazolam (mg)	2.93 ± 1.04	2.82 ± 1.01	0.452
Etomidate(mg)	18.30 ± 1.51	18.08 ± 1.64	0.336
Sufentanil (ug)	18.26 ± 2.88	17.49 ± 2.83	0.72
Rocuronium(mg)	49.76 ± 6.46	50.28 ± 6.12	0.579
Anesthesia maintenance drugs			
Remifentanil (mg)	0.63 ± 0.21	0.62 ± 0.18	0.755
Propofol (mg)	446.61 ± 64.25	459.39 ± 64.95	0.185
Sevoflurane end tidal concentration (%)	2.20 ± 0.30	2.25 ± 0.27	0.216
Preoperative hemoglobin (g/L)	131.83 ± 16.69	128.97 ± 14.91	0.226
Preoperative hematocrit (Hct) (%)	41.43 ± 4.79	40.36 ± 4.02	0.104
Preoperative albumin(g/L)	41.35 ± 3.64	41.16 ± 3.58	0.734
Preoperative comorbidities [n(%)]			0.698
Hypertension	19(20.65)	24(26.97)	
Diabetes mellitus	12(13.04)	9(10.11)	
Coronary heart disease	3 (3.26)	4(4.49)	
Perioperative use of vasoactive drugs [n(%)]			0.930
Ephedrine	30(32.61)	25(28.09)	
Atropine	9(9.78)	11(12.36)	
Operation time (min)	121.35 ± 32.46	130.29 ± 36.61	0.089
Anesthesia time (min)	194.63 ± 42.52	207.46 ± 47.30	0.166
Extubation time (min)	30.85 ± 7.86	27.75 ± 9.28	0.015
PACU stay time (min)	60(11.75)	55(11.50)	<0.001
Total hospital stay (d)	11(3)	11(3)	0.224

*Notes:* Variables presented as mean ± SD, median (interquartile range) or number of patients (%).

*Abbreviations:* BMI, body mass index; ASA, American Society of Anesthesiologists.

### 3.2. Primary outcomes

Group LUS displayed a significantly lower incidence of enhanced EVLW level within 30 min postoperatively than Group C (13.48% [12/89] vs. 44.57% [41/92]; absolute risk difference −31.1 percentage points; relative risk 0.30; *p* < 0.001). Postoperative hypoxemia occurred in 29 patients (31.52%) in Group C and 10 patients (11.24%) in Group LUS (absolute risk difference −20.3 percentage points; relative risk 0.36; *p* < 0.001) (Figure 3a). Moreover, EVLW severity grade distribution (‘none’, ‘mild’, and ‘moderate’) within 30 min showed a significant difference between the two groups postoperatively (*p* < 0.001). In Group C, 51 patients (55.43%) showed no EVLW increase, 30 (32.61%) had a mild increase, and 11 (11.96%) had a moderate increase; in Group LUS, 77 patients (86.52%) showed no increase, 9 (10.11%) a mild increase, and 3 (3.37%) a moderate increase. No severe cases were observed in either group (Figure 3b) (Table 2).

**Figure 3.**
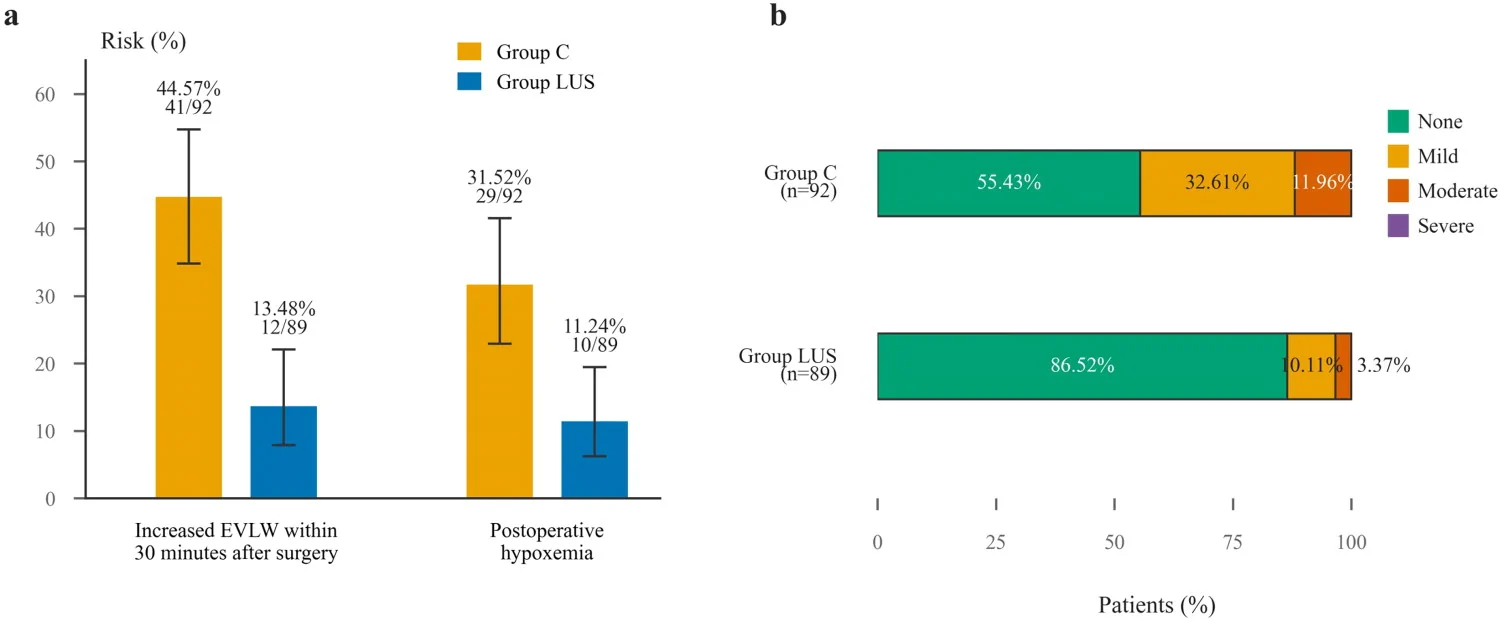
Incidence and severity of EVLW increase, and incidence of postoperative hypoxemia within 30 min after surgery.

**Table 2.** Incidence and severity of EVLW increase, and incidence of postoperative hypoxemia within 30 min after surgery.

	Group C (*n* = 92)	Group LUS (*n* = 89)	*P*-value
Incidence of increased EVLW within 30 min after surgery [n(%)]	41(44.57)	12(13.48)	<0.001
Severity of increased EVLW within 30 min after surgery [n(%)]			<0.001
None EVLW	51(55.43)	77(86.52)	
Mild EVLW	30(32.61)	9(10.11)	
Moderate EVLW	11(11.96)	3(3.37)	
Severe EVLW	0(0)	0(0)	
Incidence of postoperative hypoxemia [n(%)]	29 (31.52)	10(11.24)	<0.001

*Notes:* Variables presented as number of patients (%).

*Abbreviations:* EVLW, extravascular lung water.

### 3.3. Other secondary outcomes

Interobserver reliability for LUS scoring was high across all assessable time points. At T1, the two observers agreed on 179 of 181 assessments (163 scores of 1 and 16 scores of 2 on the diagonal), with minor disagreement in 2 cases; the weighted kappa was 0.936, indicating strong agreement. At T2 and T3, the weighted kappa was 1.000, reflecting perfect interobserver concordance. At T0, kappa could not be estimated as both observers assigned a score of 0 to all patients, leaving no score variation for analysis (Figure 4a). At T1, the score distribution in the Group C comprised 75 patients (81.52%) with a score of 1 and 17 patients (18.48%) with a score of 2, while all 89 patients (100%) in the Group LUS scored 1. No patients in either group scored 0 or 3. This distribution illustrates that the between-group difference at T1 was driven by a subset of patients in the Group C with higher LUS scores (Figure 4b). LUS scores at baseline (T0) were not significantly different in both groups (*p* > 0.05). Group LUS displayed evidently lower LUS scores at T1, T2 and T3 than Group C (*p* < 0.001) (Table 3).

**Figure 4.**
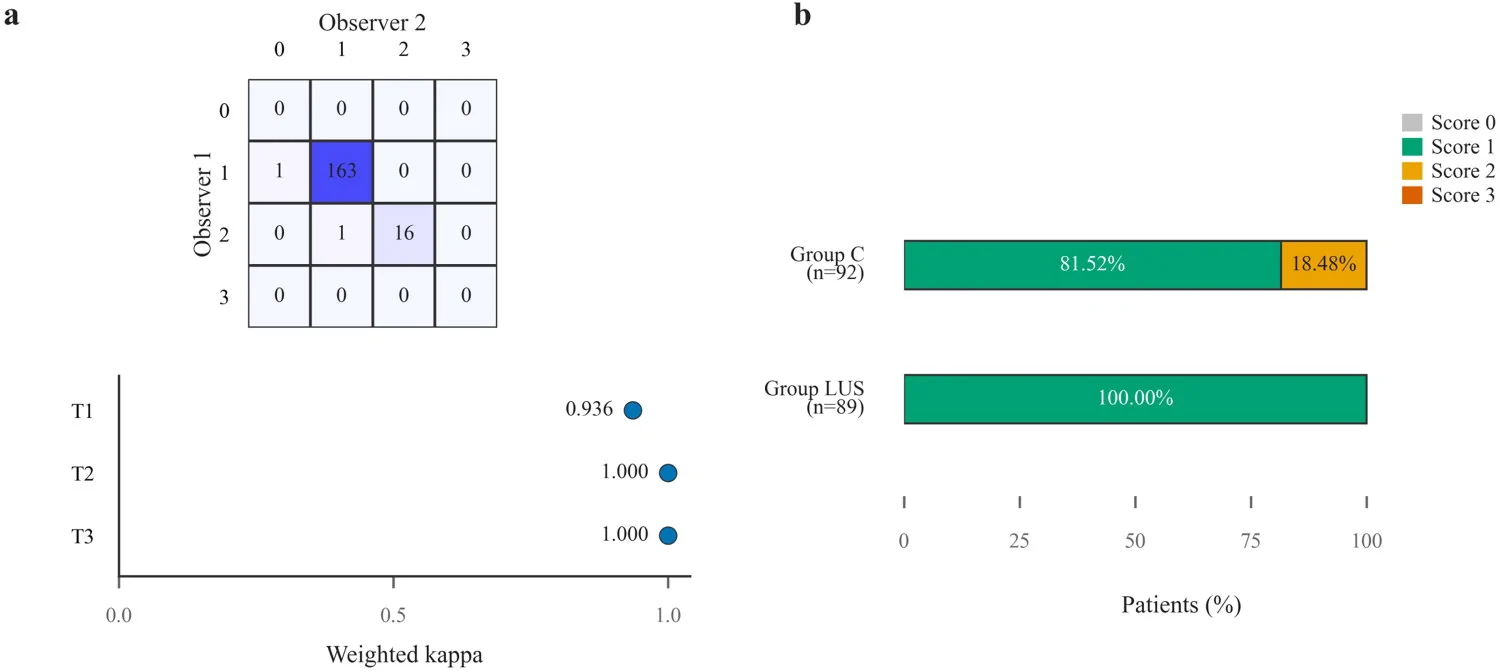
Distribution of lung ultrasound scores in the two groups and inter-observer agreement.

**Table 3.** LUS scores of both groups at various time points.

	Group C (*n* = 92)	Group LUS (*n* = 89)	*P*-value
T0	0(0)	0(0)	1.00
T1	1(0)	1(0)	<0.001
T2	1(1)	0(0)	<0.001
T3	0(1)	0(0)	<0.001

*Notes:* Variables presented as median (interquartile range).

At the baseline (T0), there was no statistically significant difference in CVP between the two groups of patients (*p* > 0.05). At T1、T2 and T3, the CVP in the Group LUS was significantly lower than that in the control group (*p* < 0.001) (Figure 5).

**Figure 5.**
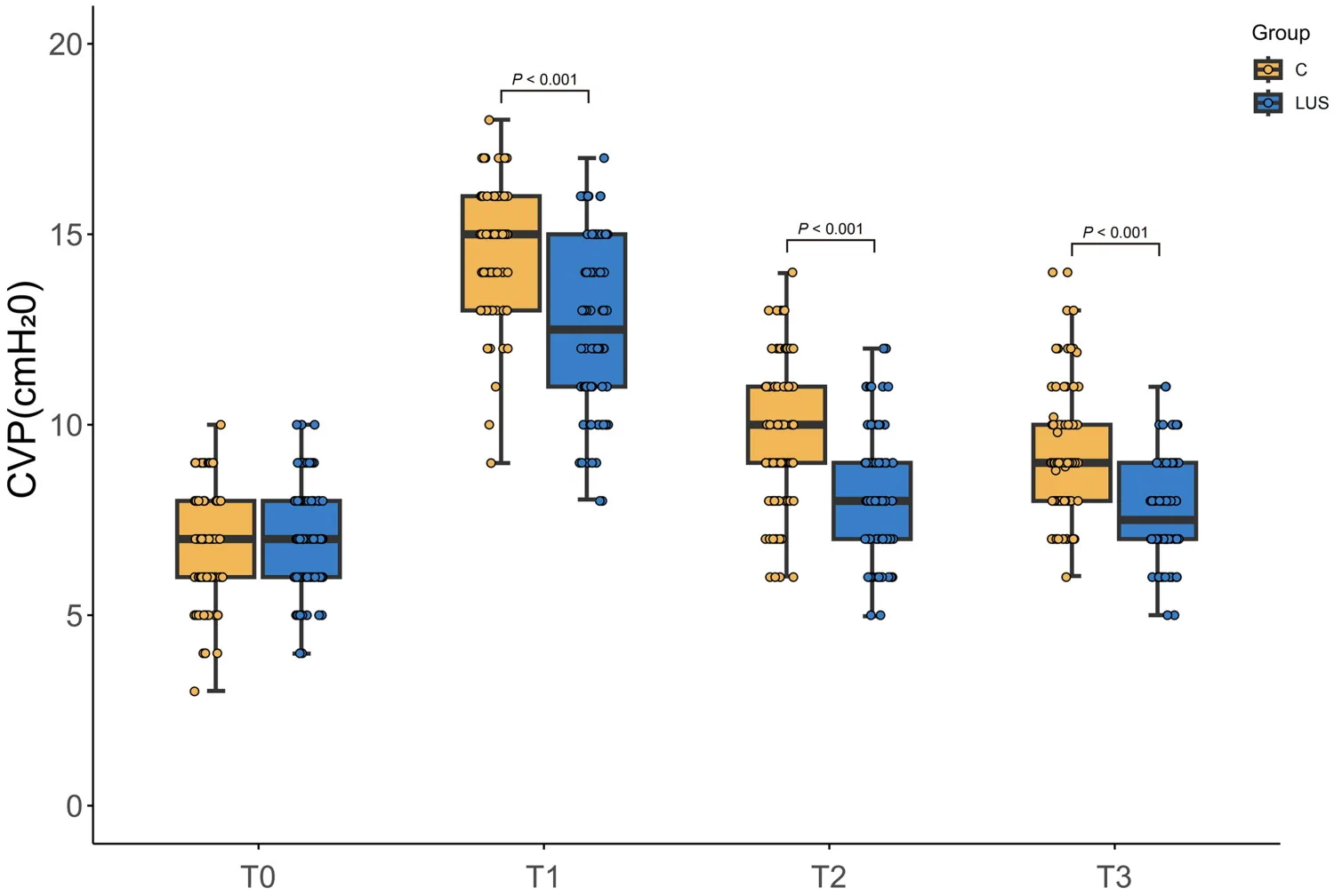
Comparison of CVP across different time points.

Differences in volumes of crystalloid infusion, colloid infusion, total fluid infusion, total irrigation volume, and fluid replacement volume at the initial administration of furosemide showed no statistical significance in both groups (*p* > 0.05). Nonetheless, irrigation volume at the first furosemide administration of Group C was markedly higher than Group LUS (*p* < 0.05). Additionally, the total furosemide dose administered increased in Group LUS relative to Group C (*p* < 0.001) (Table 4).

**Table 4.** Fluid volumes in two groups.

	Group C (*n* = 92)	Group LUS (*n* = 89)	*P*-value
Irrigation volume at first furosemide administration (ml)	15550(6000)	13300(6550)	0.002
Fluid volume at first furosemide administration (ml)	730(172.5)	740(175)	0.675
Total dose of furosemide (mg)	10(10)	10(10)	<0.001
Crystalloid volume (ml)	1000(325)	750(250)	0.085
Colloid volume (ml)	500(250)	500(250)	0.948
Total fluid infusion volume (ml)	1250(400)	1250(500)	0.239
Total irrigation volume (ml)	19575(10170)	18900(13000)	0.738

*Notes:* Variables presented as median (interquartile range).

Incidences of perioperative adverse events, like hypotension, hypertension, bradycardia, tachycardia, hypothermia, postoperative shivering, and pulmonary edema, showed no significant differences in both groups (*p* > 0.05) (Table 5).

**Table 5.** Incidences of perioperative adverse events.

	Group C (*n* = 92)	Group LUS (*n* = 89)	*P*-value
Hypotension	21(22.83)	24(26.97)	0.519
Hypertension	5(5.43)	7(7.87)	0.511
Bradycardia	21(22.83)	16(17.89)	0.419
Tachycardia	7(7.61)	6(10.11)	0.821
Hypothermia	21(22.83)	15(16.85)	0.314
Postoperative shivering	9(9.78)	8(8.99)	0.855
Pulmonary edema	4(4.35)	1(1.11)	0.186

*Notes:* Variables presented as number of patients (%).

## 4. Discussion

Renal calculi are a common urinary system disease, affecting 5% to 12% of the global population [16,17]. As the gold standard surgery for managing complex renal calculi, PCNL is advantageous in its minimal invasiveness and a great stone clearance rate [18]. Although it achieves a success rate exceeding 90%, the incidence of complications is over 10% [19], among which absorption of irrigating fluid is a common complication of PCNL [20]. Malik [21] confirmed that 78% of patients undergoing PCNL experience significant absorption of irrigating fluid. The absorption volume increases with prolonged perfusion time, thereby causing circulatory overload, oxygenation dysfunction, and acid-base imbalance. Moreover, the increased systemic blood volume caused by irrigating fluid absorption elevates the pulmonary capillaries’ hydrostatic pressure through pulmonary circulation. According to Starling’s law, this promotes fluid extravasation from capillaries into the pulmonary interstitium and alveoli, thereby enhancing EVLW level and causing pulmonary edema.

LUS has become an important tool for diagnosing pulmonary edema due to its high sensitivity and specificity (97% and 98%), respectively [22]. Studies have confirmed that LUS B-lines can serve as a semi-quantitative indicator for evaluating EVLW; an increase in B-lines is linearly related to an increase in EVLW [11]. When the EVLW level increases, the fluid in the pulmonary interstitium and alveoli increases, thereby altering the acoustic properties of lung tissue. Ultrasonography shows the appearance and increase of B-lines [23], and their quantity changes dynamically with the fluctuation of lung water volume. Additionally, the LUS scoring method quantifies the score by counting B-line positive areas in various regions of the lung, and its dynamic monitoring can help understand the changing trends of lung fluid [24].

In our study, Group LUS showed a significantly decreased incidence of enhanced EVLW level and severity within 30 min postoperatively relative to Group C. Additionally, Group LUS also exhibited decreased LUS scores and CVP relative to Group C at the time of the first administration of furosemide (T1), after irrigation (T2), and when leaving the PACU (T3). These outcomes reflect the efficacy of the comprehensive LUS-led management protocol rather than LUS monitoring alone. The advantages of the LUS group come from two complementary aspects: earlier detection of EVLW elevation before hemodynamic changes, and subsequent standardized threshold-driven interventions. Since LUS was applied as part of a complete management system, we cannot distinguish their individual contributions. This protocol enables real-time and accurate EVLW evaluation and early detection of irrigation fluid absorption.

By adopting the 28-intercostal space technique, bedside LUS can initiate intervention by identifying mild EVLW changes through B-lines when irrigating fluid absorption has not yet caused obvious hemodynamic fluctuations (CVP remains within the normal range of 5–12 cmH_2_O). This ‘advanced monitoring’ feature effectively compensates for the limitations of traditional CVP monitoring. For example, CVP reflects right ventricular preload and is susceptible to the patient’s body position [25], which may lead to ‘false normal’ judgments, i.e. EVLW actually increases, but CVP still shows normal values. Conversely, LUS is not affected by body position if the acoustic properties of lung tissue are assessed directly. Its semi-quantitative ECS maintains a high level of correlation with EVLW even in tilted positions. Thus, this might be a key reason why the monitoring accuracy of Group LUS is superior to that of Group C.

In terms of intervention timing and effect, the irrigation volume at the initial furosemide administration was significantly higher in Group C than in Group LUS, while the total furosemide dose was markedly lower in the control group. Irrigation volume and operative time are important factors that should be carefully considered, as prolonged surgical duration may be associated with increased irrigation fluid exposure and a higher risk of extravascular lung water accumulation. Although EVLW is a surrogate marker, its clinical relevance lies in its ability to identify subclinical pulmonary fluid accumulation before overt respiratory compromise develops. Excessive absorption of irrigation fluid during PCNL may impair pulmonary gas exchange and increase the risk of postoperative hypoxemia and pulmonary complications. The observed differences in fluid management suggest that the LUS-guided protocol facilitates earlier intervention. Once a mild rise in EVLW is identified *via* LUS scoring, diuretic administration and fluid restriction can halt further EVLW elevation and prevent volume overload induced by excessive absorption of irrigation fluid. In contrast, Group C only delivered interventions after cumulative irrigation fluid increased or CVP became abnormal. At this stage, elevated EVLW had already impaired pulmonary function. Moreover, the incidence of postoperative hypoxemia was significantly lower in Group LUS, which further verifies that the LUS-guided management protocol effectively mitigates postoperative EVLW elevation and associated complications. Traditional CVP-based approaches lack such real-time monitoring capacity, precluding effective intervention before complications arise. Future studies should further investigate whether this strategy can reduce postoperative monitoring requirements, pulmonary complications, and healthcare resource utilization.

We also found that the PACU stay time in Group LUS significantly reduced relative to Group C, while extubation time was significantly enhanced than Group C. Therefore, LUS reduces the interference induced by pulmonary interstitial fluid on oxygenation function by accurately controlling EVLW [26]. Increased EVLW content reduces lung compliance and increases the risk of ventilation/perfusion ratio imbalance [27]. However, the Group LUS maintained EVLW at a low level through early intervention, thereby enabling the patients’ postoperative pulmonary oxygenation function to recover more quickly, as well as shortening the extubation and PACU stay time, respectively.

## 5. Limitations

However, our study had a few limitations. First, the statistical power of our results could have been affected due to our single-center design and modest sample size. Thus, future research should conduct multicenter, large-sample randomized controlled trials, involving hospitals of different regions and levels, to verify the efficacy of LUS under different medical levels and improve our findings’ external validity. Second, we focused on the LUS application during PCNL. Our future research direction might focus on the LUS application effect in other surgeries, aiming to verify its feasibility and advantages in diverse surgical scenarios. Third, this study compared two distinct fluid management protocols rather than isolated monitoring modalities. Consequently, the observed clinical benefits cannot be attributed solely to LUS as a diagnostic tool; they reflect the combined effect of enhanced EVLW detection sensitivity and earlier, protocolized therapeutic intervention. Future studies incorporating arms with LUS monitoring alone (without protocolized intervention) or CVP monitoring with matched early intervention thresholds would be needed to isolate the independent contribution of each component. Fourth, the primary outcome involves a degree of circularity: LUS was used both to guide intraoperative intervention and evaluate therapeutic efficacy. This represents an inherent limitation of biomarker-guided trials, which may amplify between-group differences in borderline cases with mild-to-moderate EVLW. Finally, the follow-up duration should be extended to evaluate whether LUS-guided fluid management can reduce the long-term pulmonary complication rate postoperatively, thereby further expanding the long-term clinical value of the research.

## 6. Conclusions

In conclusion, an LUS-guided fluid management protocol, integrating real-time EVLW assessment with predefined intervention thresholds, demonstrated remarkable advantages over conventional CVP-based management during the perioperative period of PCNL. The sensitivity of LUS for detecting early EVLW elevation, combined with protocolized timely intervention, helps reduce the incidence of postoperative hypoxemia, shorten extubation and patients’ PACU stay time, improve short-term perioperative recovery parameters.

## Supplementary Material

CONSORTchecklist.pdf

## Data Availability

All data generated or analyzed during this study were included in the published article. Further inquiries about the datasets can be directed to the corresponding author on reasonable request.

## References

[1] Tzelves L, Türk C, Skolarikos A. European association of urology urolithiasis guidelines: where are we going? Eur Urol Focus. 2021;7(1):34–38. doi: 10.1016/j.euf.2020.09.011. 33011151

[2] Ghani KR, Andonian S, Bultitude M, et al. Percutaneous nephrolithotomy: update, trends, and future directions. Eur Urol. 2016;70(2):382–396. doi: 10.1016/j.eururo.2016.01.047. 26876328

[3] Eichhorn V, Maerz A, Salomon G, et al. Hemodynamic effects of orally administered delta-ALA during radical prostatectomy. World J Urol. 2013;31(2):371–376. doi: 10.1007/s00345-011-0788-7. 22120180

[4] Kothiyal M, Kumar N, Khurana G. Perioperative complications in percutaneous nephrolithotomy: predictive risk factors and hemodynamic alterations. Cureus. 2024;16(9):e69488. doi: 10.7759/cureus.69488. PMC11480458 39416589

[5] Lloyd‐Donald P, Fujino M, Waldman B, et al. Measurement and interpretation of central venous pressure: a narrative review. Anaesthesia. 2025;80(9):1093–1102. doi: 10.1111/anae.16633. PMC12351226 40457939

[6] Pesenti A, Slobod D, Magder S. The forgotten relevance of central venous pressure monitoring. Intensive Care Med. 2023;49(7):868–870. doi: 10.1007/s00134-023-07101-z. 37294343

[7] Assaad S, Kratzert WB, Shelley B, et al. Assessment of pulmonary edema: principles and practice. J Cardiothorac Vasc Anesth. 2018;32(2):901–914. doi: 10.1053/j.jvca.2017.08.028. 29174750

[8] Lindow T, Quadrelli S, Ugander M. Noninvasive imaging methods for quantification of pulmonary edema and congestion: a systematic review. JACC Cardiovasc Imag. 2023;16(11):1469–1484. doi: 10.1016/j.jcmg.2023.06.023. 37632500

[9] Assaad S, Kratzert WB, Perrino AC. Extravascular lung water monitoring for thoracic and lung transplant surgeries. Curr Opin Anaesthesiol. 2019;32(1):29–38. doi: 10.1097/ACO.0000000000000683. 30507676

[10] Beshara M, Bittner EA, Goffi A, et al. Nuts and bolts of lung ultrasound: utility, scanning techniques, protocols, and findings in common pathologies. Crit Care. 2024;28(1):328. doi: 10.1186/s13054-024-05102-y. PMC11460009 39375782

[11] Mongodi S, De Luca D, Colombo A, et al. Quantitative lung ultrasound: technical aspects and clinical applications. Anesthesiology. 2021;134(6):949–965. doi: 10.1097/ALN.0000000000003757. 33819339

[12] Grune J, Beyhoff N, Hegemann N, et al. From bedside to bench: lung ultrasound for the assessment of pulmonary edema in animal models. Cell Tissue Res. 2020;380(2):379–392. doi: 10.1007/s00441-020-03172-2. PMC7210222 32009189

[13] Picano E, Frassi F, Agricola E, et al. Ultrasound lung comets: a clinically useful sign of extravascular lung water. J Am Soc Echocardiogr. 2006;19(3):356–363. doi: 10.1016/j.echo.2005.05.019. 16500505

[14] Agarwal R, Toto RD, Weir MR. Extravascular lung water assessment by ultrasound to guide dry weight changes: ready for prime time? Am J Kidney Dis. 2020;75(1):1–3. doi: 10.1053/j.ajkd.2019.09.004. 31732236

[15] Picano E, Pellikka PA. Ultrasound of extravascular lung water: a new standard for pulmonary congestion. Eur Heart J. 2016;37(27):2097–2104. doi: 10.1093/eurheartj/ehw164. PMC4946750 27174289

[16] Walter K. Kidney stones. JAMA. 2022;328(9):898. doi: 10.1001/jama.2022.12609. 36066517

[17] Tan S, YUan D, Su H, et al. Prevalence of urolithiasis in China: a systematic review and meta-analysis. BJU Int. 2024;133(1):34–43. doi: 10.1111/bju.16179. 37696625

[18] Assimos D, Krambeck A, Miller NL, et al. Surgical management of stones: American urological association/endourological society guideline, PART I. J Urol. 2016;196(4):1153–1160. doi: 10.1016/j.juro.2016.05.090. 27238616

[19] Turna B, Nazli O, Demiryoguran S, et al. Percutaneous nephrolithotomy: variables that influence hemorrhage. Urology. 2007;69(4):603–607. doi: 10.1016/j.urology.2006.12.021. 17445632

[20] De S, Autorino R, Kim FJ, et al. Percutaneous nephrolithotomy versus retrograde intrarenal surgery: a systematic review and meta-analysis. Eur Urol. 2015;67(1):125–137. doi: 10.1016/j.eururo.2014.07.003. 25064687

[21] Malik I, Wadhwa R. Percutaneous nephrolithotomy: current clinical opinions and anesthesiologists perspective. Anesthesiol Res Pract. 2016;2016(1):9036872. doi: 10.1155/2016/9036872. PMC4826713 27110239

[22] Wang Y, Shen Z, Lu X, et al. Sensitivity and specificity of ultrasound for the diagnosis of acute pulmonary edema: a systematic review and meta-analysis. Med Ultrason. 2018;1(1):32–36. doi: 10.11152/mu-1223. 29400365

[23] Volpicelli G, Skurzak S, Boero E, et al. Lung ultrasound predicts well extravascular lung water but is of limited usefulness in the prediction of wedge pressure. Anesthesiology. 2014;121(2):320–327. doi: 10.1097/ALN.0000000000000300. 24821071

[24] Smit MR, Hagens LA, Heijnen NFL, et al. Lung ultrasound prediction model for acute respiratory distress syndrome: a multicenter prospective observational study. Am J Respir Crit Care Med. 2023;207(12):1591–1601. doi: 10.1164/rccm.202210-1882OC. PMC10273105 36790377

[25] Roger C, Muller L, Riou B, et al. Comparison of different techniques of central venous pressure measurement in mechanically ventilated critically ill patients. Br J Anaesth. 2017;118(2):223–231. doi: 10.1093/bja/aew386. 28100526

[26] Diwakar A, Punetha P, Kamat A, et al. EVLW by lung ultrasound to predict short-term post operative outcomes in pediatric cardiac surgery-a prospective observational study. Ann Card Anaesth. 2025;28(3):305–309. doi: 10.4103/aca.aca_230_24. PMC12324780 40629787

[27] Castagna O, Hamar V, Schmid B, et al. Physiological impact of controlled emergency swimming ascents training in professional divers: evidence of subclinical lung stress. J Appl Physiol (1985). 2025;139(2):594–603. doi: 10.1152/japplphysiol.00462.2025. 40695619

